# A Double-Headed Physiologic Monster: A Case Report and Literature Review

**DOI:** 10.7759/cureus.44362

**Published:** 2023-08-30

**Authors:** Valentine Ebuh, Juan Avila

**Affiliations:** 1 Internal Medicine, Methodist Health System, Dallas, USA; 2 Internal Medicine, Methodist Dallas Medical Center, Dallas, USA

**Keywords:** incidentaloma, ectopic acth production, endocrine oncology, adrenal glands, adrenal pheochromocytoma

## Abstract

Adrenal incidentaloma (AI) is rare and found in approximately 2-4% of abdominal computed tomography scans. Up to 10% of patients with AI have autonomous secretion of adrenal hormones. If not quickly diagnosed and adequately treated, the outcome may be devastating to the patient. On very rare occasions, a pheochromocytoma may, in addition to the production of catecholamine, produce adrenocorticotropic hormone causing Cushing disease. We present a case of a patient with pheochromocytoma and Cushing syndrome.

## Introduction

Admitted patients frequently undergo abdominal imaging for conditions other than a concern for adrenal pathology. Some of the abdominal imaging may find an incidentaloma. The latter may involve any abdominal site including an adrenal gland. When found, an incidentaloma needs proper management to reduce related morbidity and mortality. If not quickly diagnosed and adequately treated, the outcome may be devastating to the patient. A pheochromocytoma is one of the incidentalomas reported in clinical practice. On very rare occasions, a pheochromocytoma may, in addition to producing catecholamine, produce adrenocorticotropic hormone (ACTH) and cause Cushing syndrome. The adrenal medulla is physiologically related to the sympathetic nervous system and produces catecholamine in response to sympathetic stimuli. Pheochromocytoma, a tumor of the adrenal medulla, usually produces catecholamines while the adrenal cortical section generally secretes corticosteroids (i.e., mineralocorticoids and glucocorticoids) and a negligible amount of sex hormones. The adrenal cortex’s zona fasciculata is controlled by the hypothalamic-pituitary axis. The hypothalamus produces corticotrophin-releasing factor, which regulates ACTH secretion from the pituitary. ACTH then provides feedback to the zona fasciculata to adjust its secretion of cortisol. Overstimulation of this zone may cause adrenal cortical hyperplasia. Cortisol feeds back to the hypothalamus and the anterior pituitary to regulate the latter’s secretion of ACTH. ACTH may also be produced in other endogenous sites during illnesses, such as small-cell lung cancer, pancreatic cancer, medullary thyroid cancer, and pheochromocytoma. Although the functionality of the adrenal medulla and the adrenal cortex are not related and are influenced by unrelated mechanisms, a pheochromocytoma may occasionally stimulate the cortex. Pheochromocytoma may produce ectopic ACTH which stimulates the cortex to produce glucocorticoid and a small amount of androgens. Oversecretion of cortisol may lead to Cushing syndrome. Here, we present the case of a patient with pheochromocytoma and Cushing syndrome originating from a tumor of the adrenal medulla.

## Case presentation

A 66-year-old male with a past medical history of hypertension, stage 2 chronic kidney disease, and type 2 diabetes mellitus presented to the emergency department (ED) complaining of ongoing dysuria and urinary urgency for at least five days. He thought that his symptoms were due to a urinary tract infection. He used a prescribed antibiotic but could not remember the name. His family members advised that he seek medical attention as his symptoms failed to improve with the antibiotic. Associated symptoms included headache, sweats, chills, low back pain, and a fever of up to 38.7°C. These symptoms were alleviated after a single dose of 400 mg ibuprofen. He denied any weight loss, swollen glands, or enlarged lymph nodes. He denied any smoking history or illicit drug use but drank alcohol socially. His past surgical history included a cholecystectomy, and his family history consisted of his father who had an unspecified heart disease. The patient managed his diabetes mellitus with a subcutaneous insulin pump and 1,000 mg of metformin twice a day (BID). His home medicines for his hypertension included metoprolol succinate (25 mg every day (QD)), amlodipine (10mg QD), losartan (100 mg QD) and hydrochlorothiazide (25 mg QD). He was allergic to lisinopril, which caused a chronic dry cough.

In the ED, his blood pressure was 174/88 mmHg, his pulse was 110 beats per minute, his temperature was 36.7°C, and his respiratory rate was 18 breaths per minute with an oxygen saturation of 94% on room air. A physical examination was normal except for the presence of a palpable abdominal wall subcutaneous insulin pump. His initial laboratory results are summarized in Table 1 and Table 2.

**Table 1:** Complete blood count and blood chemistry. ALT: alanine transferase; AST: aspartate aminotransferase; ALP: alkaline phosphatase; BUN: blood urea nitrogen; eGFR: estimated glomerular filtration rate; MCH: mean corpuscular hemoglobin; MCHC: mean corpuscular hemoglobin concentration; MCV: mean corpuscular volume; RBC: red blood cells; RDW-CV: red cell distribution width; WBC: white blood cells

Variable	Value	Reference range
WBC (×10^3^/µL)	20.6 (H)	3.8–10.6
RBC (×10^6^/µL)	4.57	4.50–5.90
Hemoglobin (g/dL)	13.7	13.5–17.5
Hematocrit (%)	39.4 (L)	40.2–52.0
MCV (fL)	86.2	80.0–100.0
MCH (pg)	30.0	26.0–34.0
MCHC (g/dL)	34.8	31.1–36.6
RDW-CV (%)	14.3	11.5–14.5
Platelet Count (×10^3^/µL)	375	130–400
Lymphocytes (%)	2.2 (L)	24.0–44.0
Monocytes (%)	14.6 (H)	0.1–10.0
Eosinophils (%)	0.0	≤5.0%
Basophils (%)	0.1	≤2.0%
Neutrophils (×10^3^/µL)	17.0 (H)	1.4–7.3
Lymphocytes (×10^3^/µL)	0.5 (L)	0.9–4.8
Monocyte absolute count (×10^3^/µL)	3.0 (H)	0.1–1.1
Sodium (mmol/L)	131 (L)	135–148
Potassium (mmol/L)	3.6	3.4–5.1
Chloride (mmol/L)	89 (L)	95–106
CO_2 _(mmol/L)	31	22–31
Anion gap (mEq/L)	11	8–16
BUN (mg/dL)	33 (H)	10–25
Creatinine (mg/dL)	0.90	0.70–1.40
Glucose (mg/dL)	169 (H)	70–110
Calcium (mg/dL)	9.3	8.4–10.2
AST (U/L)	84 (H)	8–42
ALT (SGPT) (U/L)	86 (H)	<50
Alkaline phosphatase (U/L)	108	38–126
Total protein (g/dL)	8.3 (H)	6.0–8.0
Albumin (g/dL)	4.3	3.5–5.7
Total bilirubin (mg/dL)	1.0	0.0–1.4
eGFR (mL/minute/1.73 m^2^)	>60.0	>60.0
Corrected calcium (mg/dL)	9.1	8.4–10.2
BUN/Creatinine ratio (mg/dL)	36.67 (H)	12.00–20.00
Osmolality (mOsm/kg)	265	261–280

**Table 2:** Urine laboratory results (clean-catch, midstream). HPF: high-power field; RBC: red blood cells; WBC: white blood cells

Variable	Value	Reference range
Appearance	Clear	Clear
Bilirubin	Negative	Negative
Ketones (mg/dL)	Negative	Negative
Specific gravity (UA)	1.015	1.000–1.025
Blood	Negative	Negative
pH	5.5	5.0–8.0
Protein (mg/dL)	30 (A)	Negative
Urobilinogen (mg/dL)	0.20	0.2–1.0
Nitrite	Negative	Negative
Leukocyte esterase	Negative	Negative
Glucose (UA) (mg/dL)	1,000 (A)	Negative
WBC (/HPF)	0–2	0–2, 2–5, none seen
RBC (/HPF)	0–2	None seen, 0–2
Squamous epithelial (/HPF)	0–2	0–2, negative
Bacteria, urine slight (A) (/HPF)	Negative /HPF	Negative
Mucus, urine (/HPF)	Negative	Negative, none seen
Hyaline casts (/HPF)	0–2	Negative, none seen
Granular casts (/HPF)	2–5	Negative, none seen

**Figure 1:**
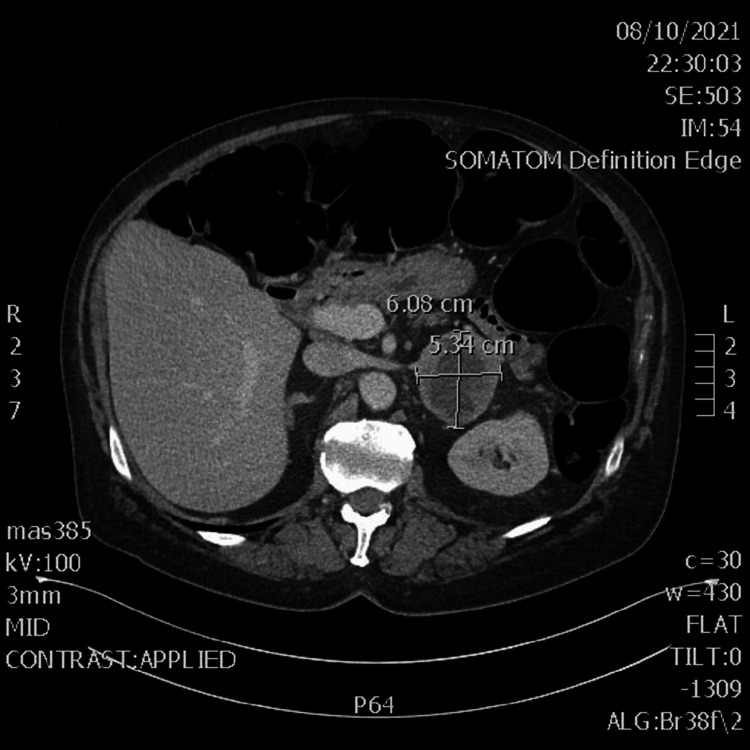
Computerized tomography scan of the abdomen and pelvis in transverse view. A computerized tomography scan of the abdomen and pelvis with intravenous contrast shows a 6.08 cm × 5.34 cm necrotic mass within the left adrenal gland.

**Figure 2:**
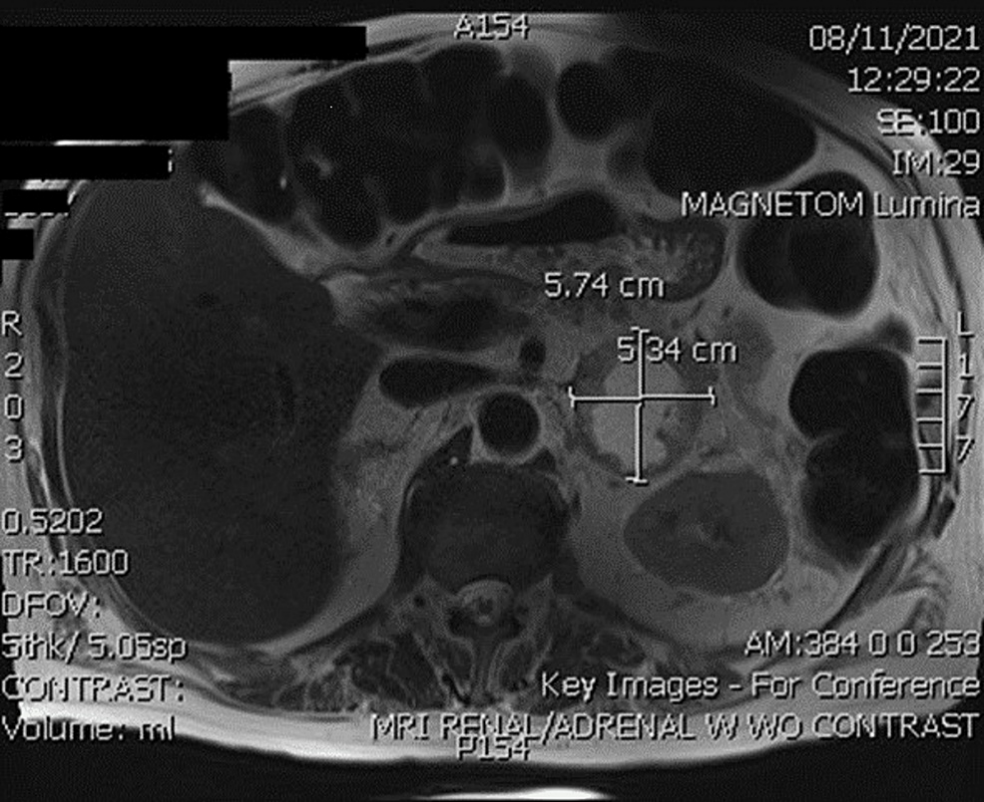
Magnetic resonance imaging transverse view. Magnetic resonance transverse view shows a 5.74 cm × 5.34 cm left adrenal mass.

**Figure 3:**
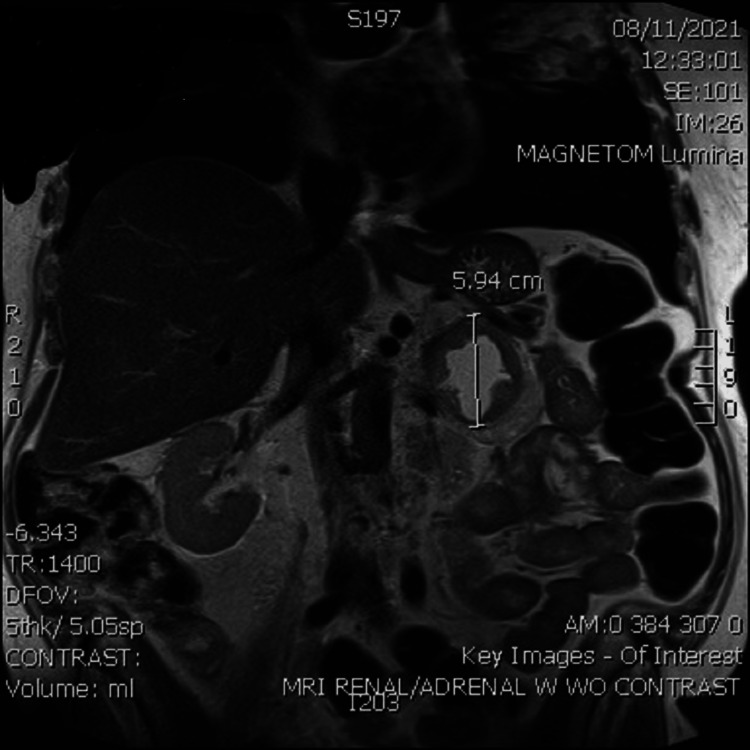
Magnetic resonance imaging showing the frontal view of the abdomen. There is a 5.94 cm mass in the left adrenal gland. There is a thickened rim of soft tissue hyperenhancement surrounding the mass.

The initial laboratory result did not elucidate a likely diagnosis and further workup was pursued. Computerized tomography (CT) imaging showed a large necrotic mass within the left adrenal gland, as shown in Figure 1. Subsequent magnetic resonance imaging (MRI) confirmed the presence of a left adrenal mass with a thickened rim enhancement concerning for malignancy, as shown in Figure 2 and Figure 3. Further laboratory workup to assess his levels of fractionated metanephrine in the plasma and serum was pursued and results are shown in Table 3. He also had a decreased thyroid-stimulating hormone level (0.39 µIU/mL) but a normal free T4 level, an elevated random urine cortisol level (81.30 µg/dL), and an increased ACTH level (376.3 pg/mL), as shown in Table 4. Given these findings and the possibility of Cushing syndrome, a pituitary gland MRI was performed; however, no lesions were identified.

**Table 3:** Fractionated metanephrine in plasma and urine.

	Variable	Value	Range
Plasma	Normetanephrine, free (nmol/L)	38.20 (H)	0.00–0.89
	Metanephrine, free (nmol/L)	1.59 (H)	0.00–0.49
Urine	Metanephrine-to-creatinine ratio (µg/g CRT)	1,522 (H)	0–300
	Normetanephrine-to-creatinine ratio (µg/g CRT)	13,583 (H)	0–400

**Table 4:** Level of hormones.

Hormones	Result	Laboratory normal range
Adrenocorticotropic hormone	376.3 pg/mL	7.2–63.3 pg/mL
Cortisol	81.30 µg/L	AM collection: 4.30–22.40 µg/dL PM collection: 3.09–16.66 µg/dL
Thyroid-stimulating hormone	0.39 µIU/mL	0.47–4.68 µLU/mL
Free T4	1.35 ng/dL	0.78–2.19 ng/dL

During the course of his admission, he had recurrent hypokalemia managed with daily oral and intravenous potassium. Nephrology was consulted to assist in managing the latter. He was also seen by the surgery team after a diagnosis of pheochromocytoma and ACTH-dependent hypercortisolemia was made. A new antihypertensive regimen, including phenoxybenzamine 60 mg BID, carvedilol 25 mg BID, amlodipine 10 mg QD, and losartan 100 mg QD was implemented to achieve the most optimal blood pressure control for the patient. He was discharged with a plan to be readmitted for left adrenalectomy in the very near future. His discharge medicines included carvedilol 25 mg BID, amlodipine 10 mg QD, losartan 100 mg QD, metformin 1,000 mg BID, phenoxybenzamine 60 mg BID, simvastatin 20 mg QD, and potassium chloride 40 mEq BID.

He was readmitted seven days later for surgery. He successfully underwent a left adrenalectomy. The pathology report confirmed a left adrenal mass favoring a benign pheochromocytoma with necrosis and background adrenal cortical hyperplasia. He was discharged in good condition on postoperative day nine. With surgical intervention, the patient was ultimately weaned off many of his blood pressure medications, only needing carvedilol 12.5 mg BID. He was to continue taking metformin 1,000 mg BID, simvastatin 20 mg QD, and insulin through his insulin pump. The patient was followed up via a telephone call five months later. At that time, he reported feeling clinically improved with normal cortisol levels. After further re-evaluation with his primary care provider, the patient no longer requires an insulin pump for diabetes management and his blood pressure remains well controlled on carvedilol 12.5 mg BID.

## Discussion

Adrenal incidentaloma (AI) is rare and found in approximately 2% to 4% of abdominal CT scans [1]. Up to 10% of AI patients have autonomous secretion of adrenal hormones [2]. Pheochromocytomas, which may be diagnosed as an incidentaloma, secrete epinephrine and norepinephrine. The tumor may continually or periodically secrete catecholamine. The production of catecholamine by this tumor plays a role in the pathogenesis of glucose regulation disorders and insulin resistance. These conditions have been shown to reverse after the resection of a pheochromocytoma [3]. Pheochromocytomas may also produce ACTH and increase cortisol secretion. When the clinical pretest probability favors further workup for pheochromocytoma, testing the urine and plasma for the levels of catecholamine and its metabolites (e.g., metanephrine) is very helpful. Tests for plasma metanephrines are more sensitive than tests for plasma catecholamine or urinary metanephrines for the diagnosis of pheochromocytoma [3,4]. Testing for metanephrine was found to have a sensitivity of 100% [5]. In addition, the negative predictive value of normal plasma concentrations of metanephrine was 100% [5]. In contrast, testing for plasma catecholamine yielded a sensitivity of 85%, and the negative predictive value of normal plasma concentrations of catecholamine was 95% [5]. Therefore, a normal plasma concentration of metanephrine eliminates the diagnosis of pheochromocytoma, but it is not excluded when the plasma concentrations of catecholamine and urinary excretion of metanephrine are normal. Our patient had both high plasma and urinary metanephrine making the diagnosis of pheochromocytoma more than likely.

Pheochromocytoma, frequently diagnosed as an incidentaloma, may remain asymptomatic for a prolonged period of time and be diagnosed incidentally. Our patient presented with unusual symptoms of dysuria and urinary urgency, which made it hard for the diagnostician to suspect pheochromocytoma. When symptoms present, they usually mimic those caused by an overactive sympathetic nervous system. This disease may be sporadic or associated with other syndromes, including multiple endocrine neoplasia type 2. Contrary to patients with the latter, our patient did not have evidence of medullary thyroid cancer or a parathyroid tumor. Pheochromocytoma generally presents as either a bilateral disease in 10% of patients or an extra-adrenal disease in 10% of patients; 10% of pheochromocytomas are malignant. Our patient had unilateral disease found on CT and MRI. Pathology of the surgically removed left adrenal gland confirmed the imaging findings.

Many patients with pheochromocytoma have diabetes and glucose intolerance. In one study, 26% had diabetes mellitus, which is consistent with the 12% to 40% incidence rate reported in other studies [3,5]. Our patient had diabetes mellitus, and his blood glucose level was not adequately controlled during his admission. His hemoglobin A1C of 7.3 is evidence of poorly controlled blood glucose levels in the preceding few months. This may be a result of both the sympathetic effect of the pheochromocytoma and ectopic ACTH production, which increased his blood cortisol level. Many pheochromocytoma patients have long-term resolution of diabetes after successful resection; however, some patients may continue to require postoperative treatment of diabetes, especially those with a higher body mass index (BMI) [6-8]. Our patient had a BMI of 29.1 kg/m^2^ before surgery. At five months post-resection, his blood sugar was better controlled and he no longer used insulin. However, he is still on metformin.

In addition to the relatively high prevalence of diabetes mellitus, patients with pheochromocytoma are more likely to have hypertension (21.8%; p < 0.001) [6-8]. Our patient had hypertension for many years, and his need for hypertension medicines was cut by more than half after resection of his pheochromocytoma.

For ACTH-dependent cortisol excess, a pituitary MRI is the investigation of choice. However, it may not show an abnormality in up to 40% of cases because small tumors are below the sensitivity of detection. Characteristically, pituitary corticotropic adenomas fail to enhance following gadolinium administration on T1-weighted MRI images [9]. When hypercortisolemia is ACTH-dependent and when the pituitary MRI is unremarkable, as was the case with our patient, further tests are needed to find the source of the ACTH. Unfortunately, no further tests to locate the source of the ACTH were done for our patient. Following the left adrenalectomy, the patient had improved blood glucose control and normalization of his cortisol levels. These findings are enough evidence to conclude that the driver of his high cortisol was not associated with pituitary ACTH production. It was likely ectopic ACTH production from a source that was removed. We think the pheochromocytoma was also producing ACTH and acting as a double-headed physiologic monster.

## Conclusions

Pheochromocytoma may have an unusual presentation and may be associated with laboratory and clinical evidence of Cushing syndrome. When a patient presents with unusual symptoms, a holistic approach may unmask unexpected diagnoses. Additionally, when a single diagnosis fails to justify some of the signs and symptoms of a patient, healthcare providers are encouraged to seek secondary diagnoses. Certain clinical conditions may be secondary to other ongoing conditions. Our patient’s diabetes mellitus and hypertension improved after the incidentaloma was treated. Sound judgment and excellent clinical acumen save lives.
